# Ilizarov Fixator-Assisted Management of Neglected Femur Fractures by Open Intramedullary Nailing: A Case Series

**DOI:** 10.7759/cureus.50864

**Published:** 2023-12-20

**Authors:** Konchada Srikant, Amar Soni, Sandeep Pradhan, Ankit Gulia, Bodanapu Sandeep, Rishab Kafley, Vigneshwaran Venkatesan, Sayashi S, Swatantra A Mohanty

**Affiliations:** 1 Orthopaedics, Kalinga Institute of Medical Sciences, Bhubaneswar, IND; 2 Orthopaedics, Soni Hospital, Dahod, IND

**Keywords:** neglected fracture, distraction osteogenesis (do), fracture non-union, femur shaft fracture, intramedullary locked nail, ilizarov ring fixator

## Abstract

Abstract: Femoral shaft fractures are major life- and limb-threatening injuries. Such injuries, when neglected for months or years, can lead to a range of debilitating consequences. In the Indian subcontinent, there are multiple such cases that are presented to tertiary care hospitals late due to a lack of awareness and low socioeconomic constraints. These neglected cases on presentation are usually in a state of nonunion or malunion, with gross shortening and deformity affecting adjacent joint mobility. The management of neglected femur shaft fractures (NFFs) demands uphill tasks, such as achieving mechanical stability, restoring length and alignment, and having strong rehabilitation schedules. The functional outcomes of these cases are always not proportional to their radiological counterparts and must be taken care of separately. In this study, intramedullary nailing of the fracture after initial distraction with the Ilizarov fixator aims to reduce deformity and shortening while restoring near-optimal functional life. The study aimed to evaluate the functional and radiological outcomes of NFFs operated with distraction by an Ilizarov fixator followed by intramedullary nailing.

Methods: Fourteen cases of NFFs presented to Kalinga Institute of Medical Sciences, Bhubaneswar, India, between January 2020 and June 2022 were recruited for the study. After preoperative evaluation and explanation of available treatment options to patients, they were operated on with a two-stage procedure of Ilizarov fixator application, followed by intramedullary interlocking nailing, with a period of gradual distraction in between. They were then followed up for a minimum of 12 months to assess functional and radiological outcomes.

Results: The average time for all of the fractures to heal was 25.2 weeks. The average knee flexion increased from 28.2 degrees before surgery to 87.1 degrees after surgery. All 14 patients could walk with complete weight bearing on the operated limb postoperatively after proper pain control measures were taken. The mean Tegner Lysholm knee score was 77.8. There was residual limping in six of the cases, which could be attributed to muscle atrophy and/or shortening in the affected limbs. In three cases, skin blisters were formed due to the acute nature of the distraction, but they all healed with a scab and scar, otherwise uneventfully. The shortening, in 13 cases, came down to 4 cm or less, which was managed with a shoe raise. The one case with a residual 5 cm shortening had a short, limping gait, and it was attributed to an extremely overriding osteopenic femur preoperatively.

Conclusion: A two-stage operation with distraction by an Illizarov fixator followed by an intramedullary fixation provides the basic advantage of not having to excise an excessive amount of bone, which may be required in primary open reduction and intramedullary fixation. It also allows the patient to carry on his daily activities as mobilization is not restricted, which is the case in an individual to whom skeletal traction is applied. Hence, in any NFF case, this algorithm of management can be considered a frontrunner in the comprehensive management of disability and deformity.

## Introduction

Femur shaft fractures are potentially life- and limb-threatening injuries, usually after high-energy trauma [1]. The standard operative procedure is to fix these with intramedullary nailing wherever possible after primary resuscitation has been done [2]. However, even in this day and age of greater awareness, there are still instances of negligence where a patient fails to give an injury as serious as a femur fracture enough attention, whether it be for lack of knowledge or financial reasons, particularly in developing countries. These neglected cases on presentation are usually in a state of nonunion or malunion, with gross shortening and malalignment affecting adjacent joint mobility. The management of such neglected femur shaft fractures (NFFs) demands challenging tasks, such as achieving mechanical stability, restoring length and alignment, and having strong rehabilitation schedules. The typical method of treatment is the application of skeletal traction, then open reduction and internal fixation using an intramedullary interlocking nail [3,4]. Sometimes, a two-staged surgery with the removal of the callus at first and fixation in the second stage is done with a period of skeletal traction application in between [5,6]. Additional bone grafting may or may not be necessary after the final fixation. However, the shortening, overriding, and bedridden condition of such patients is a major setback to their treatment. In this study, 14 NFFs that were all at least eight months old were looked at. The first step was to distract the neglected fracture site with an Ilizarov fixator. An intramedullary interlocking nail internal fixation followed this. The anticipated advantage of such an intervention was to avoid significant shortening and reduce days of immobilization on the bed for the patient. The outcomes were expected to have a say in dictating management protocols for such NFF cases.

## Materials and methods

Fourteen cases of NFFs were presented to the Department of Orthopaedics, Kalinga Institute of Medical Sciences, Bhubaneswar, India, within a period of two and a half years, from January 2020 to June 2022. With Institutional Review Board (IRB) approval number KIIT/KIMS/IEC/058/2020, the scientific and ethical committees have approved this study. Incidentally, they were all males falling in the age group of 35-60 years and belonged to a low-socioeconomic household. They have now presented to the hospital after becoming aware of government-sponsored free healthcare services, and all are beneficiaries of those schemes. The time lapse from injury to presentation ranged from eight months to 12 years.

Five of the patients were weight-bearing over the injured limb with a limp and adjusted to daily activities, while the others were adjusted to crutches. Preoperative shortening in the affected limb ranged from 6 cm to 13 cm. Wherever there was a concern about vascularity, a CT angiography was obtained.

Surgical technique

All cases were managed in two stages. In stage 1, surgery is performed with the patient in a supine position. After anesthesia and before the incision, the knee is manipulated to get the maximum range of movement possible to get away from knee stiffness, if any. The spanning ring/Ilizarov fixator is applied with two rings or arches on either fragment across nonunion. Any bridging bone at the malunion site is osteotomized with percutaneous drill holes and an osteotome. The maximum on-table distraction is achieved across the nonunion until soft tissues are stretched and if there is a change in distal pulses. The patient is taught to distract every day in the range of 1-5 mm per day, divided equally into four intervals as per the patient’s tolerance, and a close watch is done on any changes in a distal neurovascular status. Distraction is continued until the fracture ends nearly proximally. If the patient experiences excess pain during the distraction process, then it has either slowed down or stopped temporarily and resumed after a gap. The average number of days of distraction ranges from 13 to 30 days, depending on the amount of overriding. The approximation of bone ends or the achieved distraction helps in stage 2 of surgery for easy conversion to intramedullary nailing.

**Figure 1:**
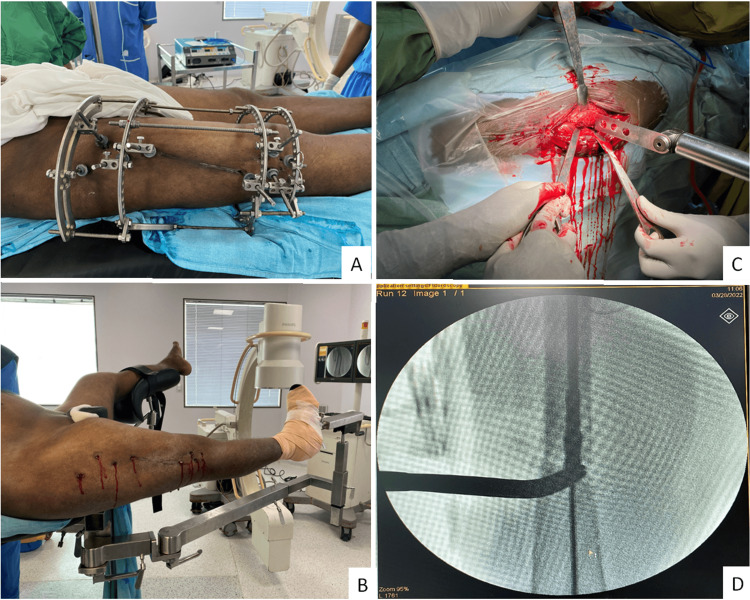
Images showing application of the Ilizarov fixator (A), after removal of the fixator (B), and open reduction and internal fixation with an intramedullary nail (C,D) A: Ilizarov fixator for distraction of fracture fragments; B: Intraoperative after removal of the ring fixator; C: Excision of fibrous tissue and open reduction; D: Intramedullary naling for final fixation

In stage 2, surgery is performed with the patient supine and attached to the fracture table. The Ilizarov fixator is removed from the table after anesthesia. Pin tracts are cleaned and taken good care of during painting and draping. The fracture site is opened, and ends are trimmed until they are approximated. The medullary cavity on both ends of the fracture was made patent through the open method, and alignment and rotation were maintained as much as possible. Standard intramedullary interlocking nailing was done (Figure 1).

In two cases, proximal femur nailing was done because of the subtrochanteric nature of the fracture. The surgeon performed autologous iliac crest bone grafting as and when he felt it was necessary. After the procedure was done, the hip and knee range of motion was checked under anesthesia, and manipulation was done before the closure of the wounds.

**Figure 2:**
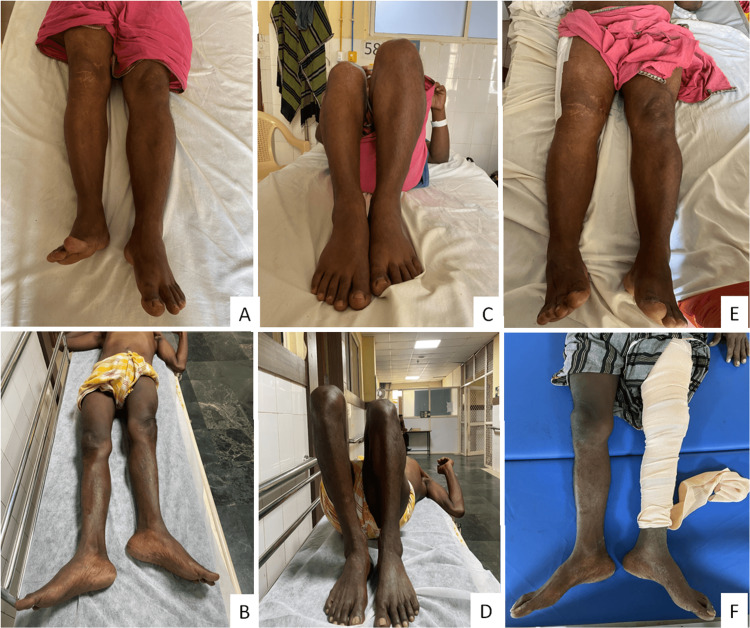
Preoperative lower limb discrepancies on knee extension (A, B) and knee flexion (C, D) and postoperative clinical photos (E, F) of patient numbers 2 and 9 show limb length discrepancies, respectively.

**Figure 3:**
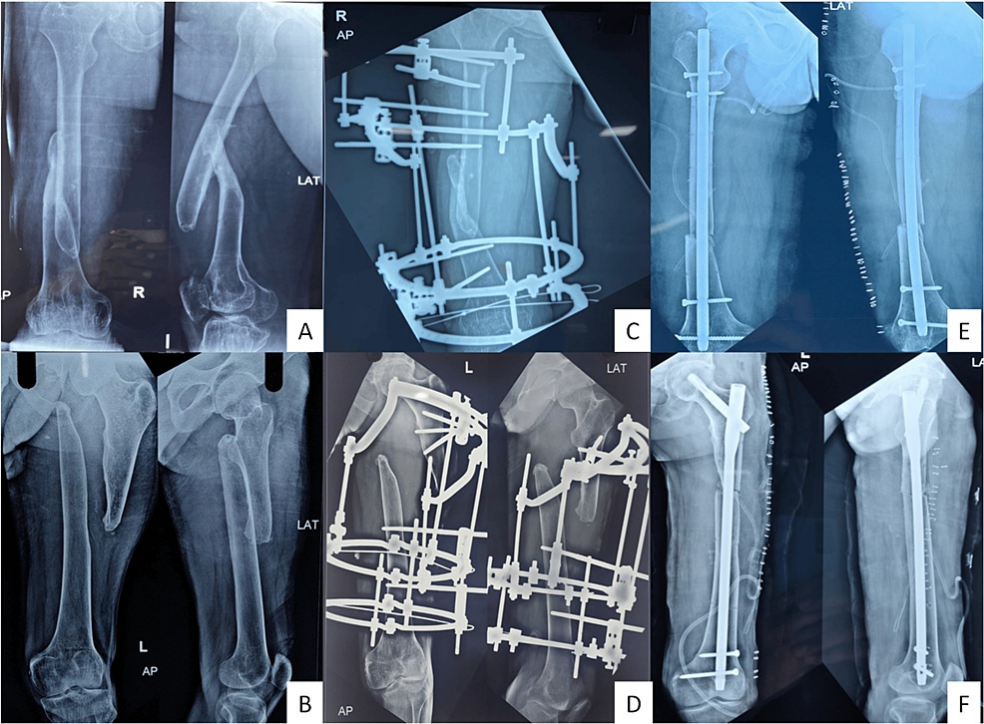
Preoperative (A, B) and postoperative radiograph with the application of the Ilizarov fixator for distraction (C, D), and following second-stage intramedullary nailing (E, F)

Postoperatively, the patients were encouraged to perform knee bending, quadriceps, hamstring, and gluteal strengthening exercises. Weight-bearing walking as tolerated was advocated from postoperative day 3. Representative preoperative and immediate postoperative clinical and radiological photos of patient numbers 2 and 9 are shown in Figure 2 and Figure 3.

## Results

The mean fracture healing time was 25.2 weeks. After surgery, the average knee flexion improved to 87.1 degrees, compared to 28.2 degrees before surgery. There were no uneventful complications intraoperatively in any of the cases. The time lapse from injury to surgery ranged from eight months to 144 months. All patients were followed up until the fractures healed, with an average follow-up of 12 months, ranging from 10 to 14 months. The mean duration of hospital stay was seven days for the first stage of treatment and five days for the second, with at least two visits averaging in between. The average diameter of the nail used was 11 mm (range, 10-12 mm). All 14 patients could weight-bear postoperatively over the affected limb after proper pain control measures were taken. The shortening, in 13 cases, came down to 4 cm or less, which was managed with a shoe raise. The mean shortening of the cases postoperatively was 3.21 cm. The one case with a 13 cm preoperative shortening and neglected for 12 years had a residual 5 cm shortening after surgery with a short-limbed limping gait, and it was attributed to his premorbid condition being a severely osteoporotic femur. Knee and hip range of movements improved satisfactorily after physiotherapy in 13 of the 14 cases, with one of them having residual knee stiffness. In eight cases, the initial range of movement decreased, but the final range of knee movement in this group was good enough for their working activities. The average arc of flexion was 87.1 degrees (range 70-120 degrees). The mean Tegner Lysholm knee score was 77.8 (Table 1).

**Table 1:** Demographic characteristics and clinical outcomes. M: male; +/-: present/absent; cm: centimeters

Patient	Age/Sex	Time lapse/Neglected for (months)	Preoperative shortening (cm)	Abnormal mobility of a fracture	Preoperative knee range of motion (degrees)	Postoperative shortening (cm)	Postoperative knee range of motion (degrees)	Tegner Lysholm knee scoring scale ( 0-100)
1	36/M	8	12	+	0-30	4	0-110	81
2	48/M	13	10	+	0-40	3	0-80	78
3	58/M	9	6	+	0-25	2	0-90	83
4	56/M	16	9	+	20-50	3	0-100	70
5	42/M	8	8	+	0-40	2	0-90	85
6	46/M	19	8	+	0-15	3	0-70	72
7	38/M	26	7	-	30-50	2	20-80	69
8	54/M	10	10	+	0-25	3	0-70	74
9	56/M	144	13	+	0-20	5	0-80	71
10	49/M	17	12	+	0-30	4	0-120	87
11	51/M	20	13	+	0-25	3	0-80	81
12	41/M	9	8	-	0-40	4	0-110	86
13	58/M	38	6	+	10-40	2	0-90	77
14	59/M	96	11	+	15-40	3	0-70	76

**Figure 4:**
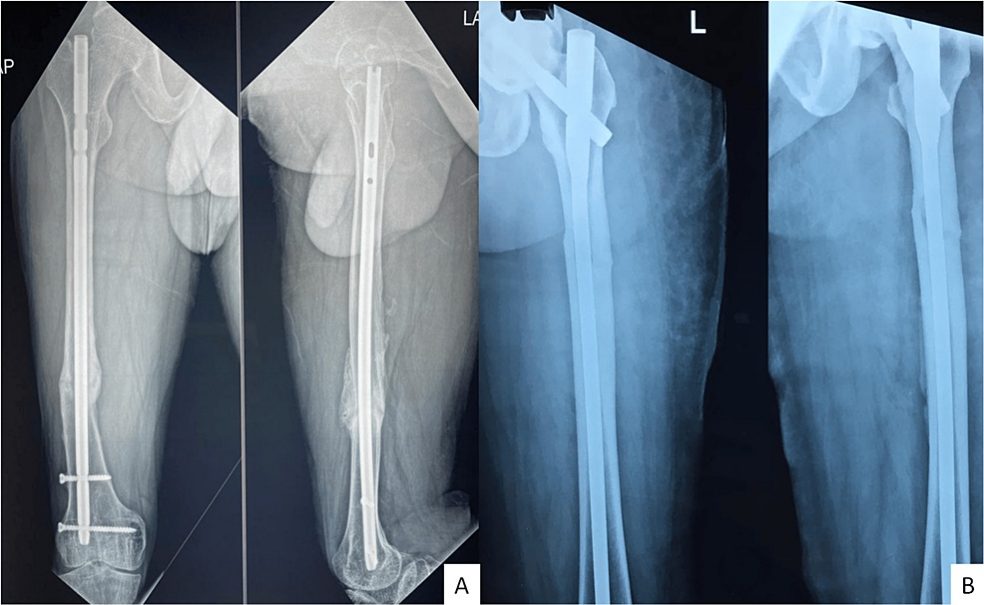
One-year postoperative X-ray of the healed fractures of patients 2 and 9.

There was some residual limping in six of the cases, which could be attributed to muscle atrophy in the affected limbs. All patients had normal alignment of the femur and the ankle and knee joints. In three cases, skin blisters were formed due to the relatively acute nature of the distraction, but they all healed with a scab and scar, otherwise uneventfully. There were no incidences of paraesthesia, foot drop, loss of sensation, or compartment syndrome in any of the managed cases. However, we were watchful for all of them because of the magnitude of the intervention being imparted. Representative one-year postoperative radiographs of patient numbers 2 and 9 are presented in Figure 4.

## Discussion

One of the most remarkable advancements in orthopaedics of the 20th century is Ilizarov's contribution [7] to the treatment of bone defects and nonunions [8]. The primary method of treatment for patients with significant bone deformities and defects is still distraction osteogenesis [9]. A gradual distraction may be able to correct deformities, such as significant limb shortening that are impossible to correct simultaneously. A lengthy postoperative period with the external fixator on the limb, however, compelled the surgeons to seek methods that would shorten that time and enhance the quality of life for their patients.

Intramedullary locking nailing is the gold standard treatment for diaphyseal long bone fractures. The medullary canal's reaming has a wide range of biological effects. One of these is a profound hyperaemic response that causes the bone cortex to revascularize [10]. Since the site of pseudoarthrosis has a limited blood supply, intramedullary reaming with nailing contributes to a better management of nonunion [11].

After analyzing all the benefits and drawbacks of the Ilizarov ring fixator and the intramedullary nailing procedure, we combined the use of both of these techniques in our study. Such a strategy for nonunion management in clinical settings sets up the atmosphere for early rehabilitation and then lets patients engage in more physical activities. In addition, when compared with the traditional Ilizarov procedure, the external fixation duration was shorter, the in-patient stay was shorter, and the postoperative recovery was quicker [12].

When fractures are neglected for months without traction, they are malaligned with shortening and overlapping of the bone fragments. One-stage open reduction is difficult due to soft tissue contracture, and it either creates acute lengthening of the shortened limb or severe shortening [13]. Serious neurovascular complications, such as scaitic nerve injury, or injury to the perineal integument and soft tissues, and iatrogenic compartment syndrome of the well leg have been reported with acute femoral lengthening performed on a shortened femur. A high incidence of complications has been reported after acute femoral lengthening of more than 6 cm [5,13].

Our study's findings are quite comparable to the results of previous research studies. The mean time to fracture union was 25.24 weeks in our studies, compared to 16 weeks in Boopalan et al.'s trial, which had 17 patients, whereas our study involved 14 patients. These differences are favorably similar [3]. Although the sample size of Akinyoola et al.'s study was quite larger than our 14 cases, the results, however, match positively with our investigation. His study had 52 individuals, with an average duration until union of 20 weeks [14,15].

This study sheds light on how a devastating injury like a femur shaft fracture, even when neglected, can be managed with a step-wise approach of patiently distracting the fracture site and freshening only the smooth, tapered ends of the bone to give a better chance at union without causing significant shortening.

The potential benefit and level of patient satisfaction in the form of a limb in alignment that produces no or a minor limp are responsible for the attractive nature of the intervention. The amount of bone freshened and resected was the least necessary to accomplish reduction. Within a few months, the majority of patients had improved their range of knee flexion. Although this study has its limitations, considering the limited number of patients handled by this algorithm, further work and documentation may well help in the growth of evidence in favor of (or against) this particular intervention.

## Conclusions

In an NFF, the patient's presentation and demands are to be properly evaluated before deciding on a management algorithm. The main benefit of a two-stage treatment with Illizarov distraction and intramedullary fixation is that it avoids the need for extensive bone excising that may be necessary for primary open reduction and intramedullary fixation. It also allows the patient to carry on his daily activities as mobilization is not restricted, contrary to the case with spinal traction-applied individuals. Hence, in any NFF case, this algorithm can be considered a frontrunner in the comprehensive management of disability and deformity.

## References

[1] Denisiuk M, Afsari A (2023). Femoral shaft fractures. StatPearls [Internet].

[2] Wood GW 2nd (2006). Intramedullary nailing of femoral and tibial shaft fractures. J Orthop Sci.

[3] Boopalan PR, Sait A, Jepegnanam TS, Matthai T, Varghese VD (2014). The efficacy of single-stage open intramedullary nailing of neglected femur fractures. Clin Orthop Relat Res.

[4] Gahukamble A, Nithyananth M, Venkatesh K, Amritanand R, Cherian VM (2009). Open intramedullary nailing in neglected femoral diaphyseal fractures. Injury.

[5] Mahaisavariya B, Laupattarakasem W (1995). Late open nailing for neglected femoral shaft fractures. Injury.

[6] Pihlajamäki HK, Salminen ST, Böstman OM (2002). The treatment of nonunions following intramedullary nailing of femoral shaft fractures. J Orthop Trauma.

[7] Ilizarov G (2012). Transosseous osteosynthesis.

[8] Marti RK, Kloen P (2011). Concepts and cases in non-union treatment.

[9] Solomon L (2012). The basic principles of external skeletal fixation using the Ilizarov and other devices, second edition. https://www.ortho-suv.org/public/Basic-Principles-of-ExFix-ProductFlyer.pdf.

[10] Klein MP, Rahn BA, Frigg R, Kessler S, Perren SM (1990). Reaming versus non-reaming in medullary nailing: interference with cortical circulation of the canine tibia. Arch Orthop Trauma Surg.

[11] Hierholzer C, Glowalla C, Herrler M, von Rüden C, Hungerer S, Bühren V, Friederichs J (2014). Reamed intramedullary exchange nailing: treatment of choice of aseptic femoral shaft nonunion. J Orthop Surg Res.

[12] Mukherjee SK, Jain V (2005). Neglected femoral diaphyseal fracture. Clin Orthop Relat Res.

[13] Paley D, Herzenberg JE, Paremain G, Bhave A (1997). Femoral lengthening over an intramedullary nail. A matched-case comparison with Ilizarov femoral lengthening. J Bone Joint Surg Am.

[14] Akinyoola L, Orekha O, Odunsi A (2011). Open intramedullary nailing of neglected femoral shaft fractures: indications and outcome. Acta Orthop Belg.

[15] Gavaskar AS, Kumar R (2010). Open interlocking nailing and bone grafting for neglected femoral shaft fractures. J Orthop Surg (Hong Kong).

